# Comparison of surgical outcomes between isolated pancreaticojejunostomy, isolated gastrojejunostomy, and conventional pancreaticojejunostomy after pancreaticoduodenectomy: a systematic review and meta-analysis

**DOI:** 10.1186/s12876-020-01415-8

**Published:** 2020-08-20

**Authors:** Yunxiao Lyu, Bin Wang, Yunxiao Cheng, Yueming Xu, Wei Bing Du

**Affiliations:** Department of Hepatobiliary Surgery, Wenzhou Medical University Affiliated Dongyang Hospital, 60 West Wuning Road, Dongyang, 322100 Zhejiang Province China

**Keywords:** Isolated Pancreaticoduodenectomy, Pancreaticojejunostomy, Gastrojejunostomy, Meta-analysis

## Abstract

**Background:**

We aimed to compare the safety and effectiveness of the following procedures after pancreaticoduodenectomy: isolated pancreaticojejunostomy, isolated gastrojejunostomy, and conventional pancreaticojejunostomy.

**Methods:**

We performed a systematic search of the following databases: PubMed, Embase, Web of Science, the Cochrane Central Register of Controlled Trials, and ClinicalTrials.gov until 1 January 2020. Pooled odds ratios (OR) or weighted mean differences (WMD) with 95% confidence intervals (CIs) were calculated using STATA 12.0 statistical software.

**Results:**

Thirteen studies involving 1942 patients were included in this study. Pooled analysis showed that reoperation rates following isolated pancreaticojejunostomy were lower reoperation than with conventional pancreaticojejunostomy (OR = 0.36, 95% CI: 0.15–0.86, *p* = 0.02, respectively), and that isolated pancreaticojejunostomy required longer operation time vs conventional pancreaticojejunostomy (WMD = 43.61, 95% CI: 21.64–65.58, *P* = 0.00). Regarding postoperative pancreatic fistula, clinically-relevant postoperative pancreatic fistula, delayed gastric emptying, clinically-relevant delayed gastric emptying, bile leakage, hemorrhage, reoperation, length of postoperative hospital stay, major complications, overall complications, and mortality, we found no significant differences for either isolated pancreaticojejunostomy versus conventional pancreaticojejunostomy or isolated gastrojejunostomy versus conventional pancreaticojejunostomy.

**Conclusions:**

This study showed that isolated pancreaticojejunostomy was associated with a lower reoperation rate, but required longer operation time vs conventional pancreaticojejunostomy. Considering the limitations, high-quality randomized controlled trials are required.

## Background

Pancreaticoduodenectomy, one of the most complex intra-abdominal operations, is widely used for benign and malignant disease located in the pancreatic head or periampullary region [1, 2]. Despite developments in surgical techniques, pancreaticoduodenectomy is still accompanied by a high postoperative complication rate of 40–50% [3]. Previous studies demonstrated that the most common complications after pancreaticoduodenectomy were postoperative pancreatic fistula (POPF) and delayed gastric emptying (DGE) [3, 4]. Several methods of digestive tract reconstruction have been recommended to reduce the main postoperative complications, namely conventional pancreaticojejunostomy, isolated pancreaticojejunostomy [5, 6], and isolated gastrojejunostomy [7, 8]. Isolated pancreaticojejunostomy, first described in 1976, was proposed to reduce complications such as POPF, based on the theory of separating bile and pancreatic enzymes [5]. In isolated gastrojejunostomy, a second loop is made to perform the gastroenteric anastomosis, which may favor digestive transit by separating the pancreatic enzymes and gastric sutures [9]. However, the debate regarding these three reconstructions is on-going. Some studies demonstrated that isolated pancreaticojejunostomy and isolated gastrojejunostomy may be associated with less postoperative complications, such as POPF [10] and DGE [11]. Kaman et al. suggested that morbidity and mortality could be reduced using isolated pancreaticojejunostomy to separate the bile from pancreatic secretions [12]; however, other studies reached different conclusions. Furthermore, studies comparing isolated pancreaticojejunostomy versus conventional pancreaticojejunostomy and isolated gastrojejunostomy versus conventional pancreaticojejunostomy involved low numbers of patients or had retrospective designs. With the recent publication of several new studies, we performed this systematic review and meta-analysis to compare the surgical outcomes of isolated pancreaticojejunostomy, isolated gastrojejunostomy, and conventional pancreaticojejunostomy.

## Methods

### Search strategy

We performed a systematic review and meta-analysis according to the Preferred Reporting Items for Systematic Reviews and Meta-Analysis (PRISMA) statement. Two authors independently searched the electronic databases of PubMed, Embase, Web of Science, Cochrane Central Register of Controlled Trials (CENTRAL), and ClinicalTrials.gov. Published trials comparing the efficacy and safety of isolated pancreaticojejunostomy, isolated gastrojejunostomy, and conventional pancreaticojejunostomy after pancreaticoduodenectomy were evaluated in this study. We used the following English search terms: “pancreaticoduodenectomy,” “pancreatoduodenectomy,” “Whipple,” “pylorus-preserving pancreaticoduodenectomy,” “pancreaticojejunostomy,” “Rou-en-Y,” and “isolated Roux loop gastrojejunostomy.” The search was restricted to human subjects, available full text, and English-language articles. The references of the articles identified after the initial search were also manually reviewed.

### Inclusion and exclusion criteria

Studies were included based on the following criteria: (1) trials had to compare isolated pancreaticojejunostomy or isolated gastrojejunostomy versus conventional pancreaticojejunostomy in patients undergoing pancreaticoduodenectomy or pylorus-preserving pancreaticoduodenectomy; and (2) complete data were provided in English with available full text. Reviews, conference abstracts, and studies with unavailable full text were excluded.

### Outcome measures

The analyzed outcome measures were POPF, clinically-relevant POPF (CR-POPF), DGE, clinically-relevant DGE (CR-DGE), operation time, bile leakage, hemorrhage, reoperation, length of postoperative hospital stay, major complications, overall complications, and mortality. The definition of POPF, CR-POPF, DGE and CR-DGE was according to the criteria of International Study Group for Pancreatic Surgery (ISGPS) [13, 14]. Major complications were defined as Clavin-Dindo grade ≥ IIIa [15]. Isolated pancreaticojejunostomy was created to prevent pancreatic fistula by separating pancreatic juice from bile juice after pancreaticoduodenectomy. Isolated gastrojejunostomy was created to prevent delayed gastric emptying after pancreaticoduodenectomy. Then, subgroup analyses were conducted depending on the different construction method.

### Data extraction and quality assessment

Data extraction was performed by two independent authors using a standardized selection form that included the first author, year of publication, type of study, country in which the study was performed, type of reconstruction, and general data. Conflicts in data abstraction were resolved by consensus and by referring to the original article. EndNote version X8 (Thomson Reuters, Toronto, ON, Canada) was used to remove duplicate studies. The methodological quality of all included studies was assessed using the validated Newcastle–Ottawa scale [16]. Studies scoring > 7 were considered of high quality.

### Statistical analysis

All statistical analyses were performed using STATA/SE 12.0 (Stata Corp., College Station, TX, USA). We used odds ratios (OR) with 95% confidence intervals (CIs) for dichotomous outcomes, and weighted mean difference (WMD) with 95% CIs for continuous variables. A two-tailed *p* value < 0.05 was considered statistically significant. Heterogeneity among studies was evaluated using the χ^2^ test; values < 25, 25–50, and > 50 were classified as low, moderate, and high heterogeneity, respectively, and were treated as binary data. We created funnel plots and performed Egger’s test [17] to evaluate the risk of publication bias. Sensitivity analyses were performed by removing individual studies from the data set and analyzing the effect on the overall results, to identify sources of signficant heterogeneity. This type of analysis is called “Jackknife analysis”, named by John Tukey, which can improvise a solution for a variety of problems [18].

## Results

### Study selection and trial characteristics

**Fig. 1:**
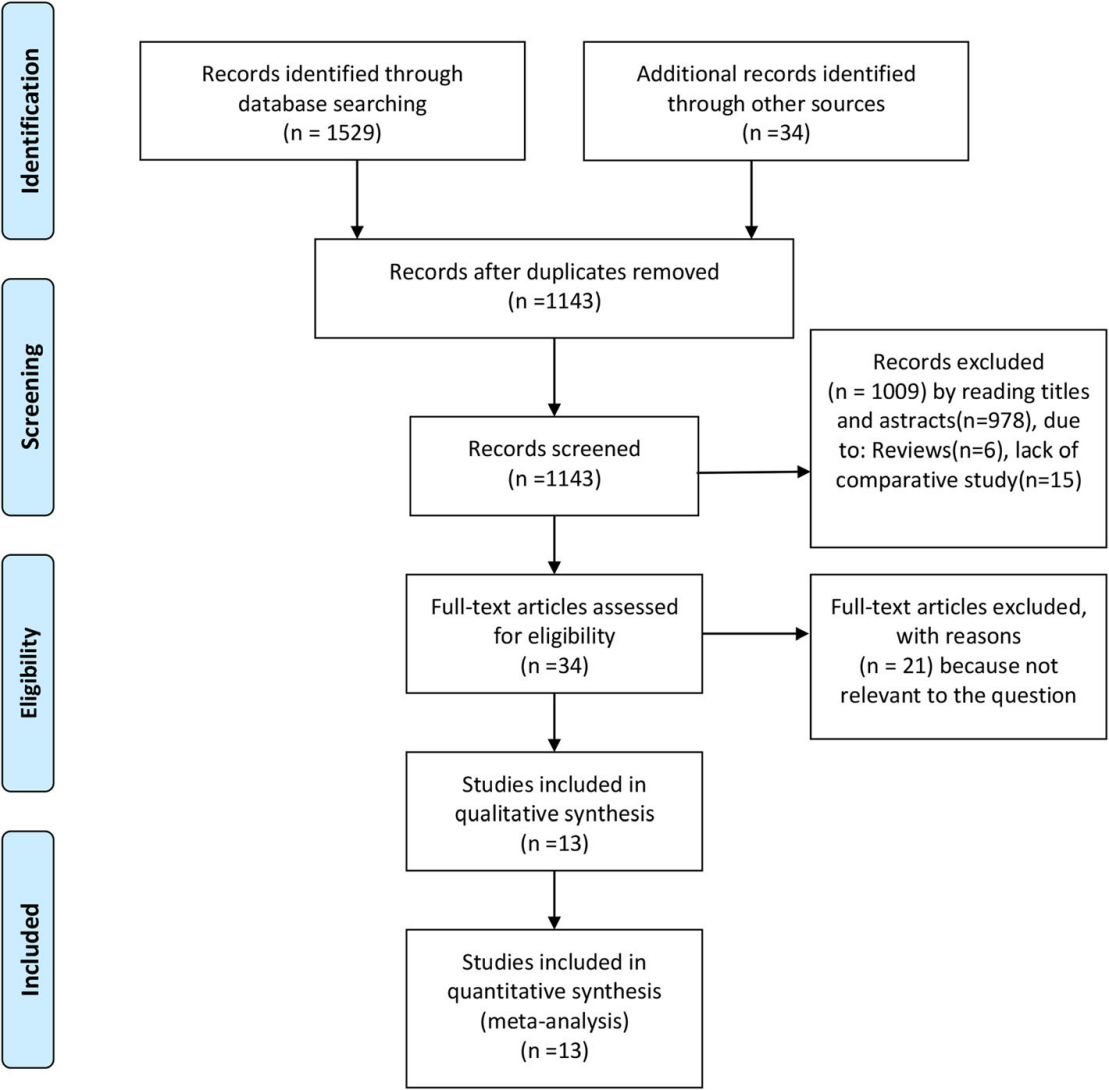
Flow diagram of the published articles evaluated for inclusion in this meta-analysis

According to the search strategy, we identified 1563 studies. Of these, 420 duplicate articles were excluded, and we retrieved the remaining 1143 studies based on their titles and abstracts. After excluding irrelevant articles for various reasons, we included a final 14 trials involving a total of 2043 patients [6, 9–12, 19–27]. A flowchart of the literature search process is shown in Fig. 1, and the characteristics and quality evaluation of the included studies are shown in Table 1. There were four randomized controlled trials (RCTs), one prospective study, and eight retrospective studies. The isolated pancreaticojejunostomy group comprised 482 patients, the isolated gastrojejunostomy group comprised 92 patients, and the isolated pancreaticojejunostomy + gastrojejunostomy group comprised 112 patients. The sample sizes among the studies ranged from 40 to 700 patients.

**Table 1:** Characteristic of the included trials

First author	Year	Country	Design	Intervention	*N*	Age	Gender (Male/Female)	PD/PPPD	NOS
Aghalarov	2018	Germany	Retro	Isolated PJ Conventional PJ	25 50	65 ± 11 64 ± 11	10/15 17/33	5/20 6/44	7
Ballas	2010	Greece	Retro	Isolated PJ Conventional PJ	46 42	64.4 ± 9.5 60.9 ± 11.5	29/17 23/19	38/8 25/17	7
Ben-Ishay	2019	Israel	Retro	Isolated GJ Conventional PJ	52 127	68.2 ± 9.6 68 ± 13.7	26/26 62/65	52/0 127/0	8
Busquets	2018	Spain	RCT	Isolated GJ Conventional PJ	40 40	68.1 ± 11.7) 65.6 ± 10.9)	24/16 24/16	40/0 40/0	9
Casadei	2008	Italy	Pro	Isolated PJ Conventional PJ	18 20	65.7 ± 10.0 56.3 ± 11.0	11/7 13/7	14/4 11/9	8
Chhaida	2018	Tunisia	Retro	Isolated PJ Conventional PJ	35 35	61 (44–74) 61 (44–74)	12,23 12,23	35/0 35/0	7
EL-SOROGY	2016	Egypt	RCT	Isolated PJ Conventional PJ	20 20	53 (29–66) 56 (34–73)	10/10 16/4	0/20 0/20	7
Grobmyer	2008	USA	Retro	Isolated PJ + GJ Conventional PJ	112 588	68 (36–83) 70 (38–90)	70/42 288/300	112/0 588/0	8
Kaman	2008	India	Retro	Isolated PJ Conventional PJ	60 51	51 ± 13.3 50 ± 13.6	39/21 35/16	60/0 42/9	8
Ke	2013	China	RCT	Isolated PJ Conventional PJ	107 109	58.3 ± 5.9 59.3 ± 6.6	51/56 50/59	107/0 109/0	8
Li	2015	China	Retro	Isolated PJ Conventional PJ	43 43	54.2 ± 9.6 53.8 ± 10.2	27/16 27/16	12/31 10/33	7
Perwaiz	2006	India	Retro	Isolated PJ Conventional PJ	53 55	53.3 ± 12.1 53.5 ± 10.1	40/13 41/14	53/0 53/0	7
Shimoda	2013	Japan	RCT	Isolated GJ Conventional PJ	49 52	65.7 ± 11.1 66.5 ± 9.8	28/21 32/20	49/0 52/0	8
Tani	2014	Japan	RCT	Isolated PJ Conventional PJ	75 76	69.6 ± 7.9 68.0 ± 8.9	39/36 42/34	2/73 7/70	8

*Retro* retrospective, *Pro* prospective, *RCT* randomized controlled trial, *PJ* pancreaticojejunostomy, *GJ* Gastrojejunostomy, *PD* pancreaticoduodenectomy, *PPPD* pylorus preserving pancreaticoduodenectomy

### Meta-analysis

#### POPF

**Fig. 2:**
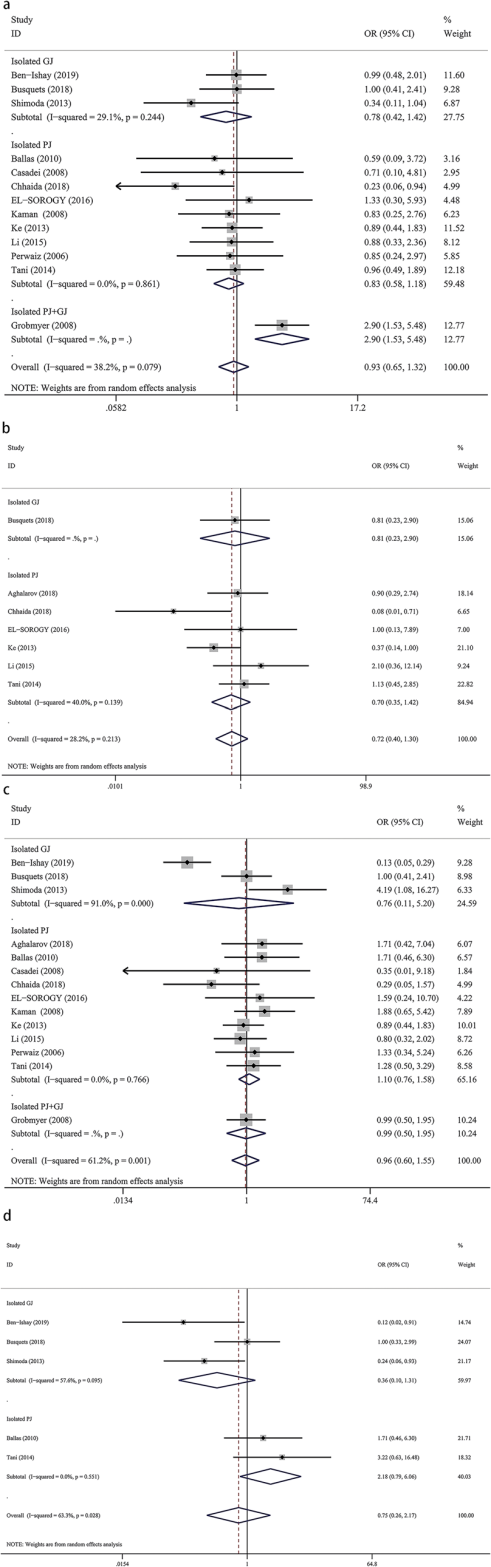
Forest plot of the meta-analysis comparing isolated pancreaticojejunostomy, isolated gastrojejunostomy, and conventional pancreaticojejunostomy regarding (**a**) postoperative pancreatic fistula, (**b**) clinically-relevant postoperative pancreatic fistula, (**c**) delayed gastric emptying, and (**d**) clinically-relevant delayed gastric emptying

Twelve studies provided data regarding POPF, with 908 patients in the isolated pancreaticojejunostomy versus conventional pancreaticojejunostomy group, 259 patients in the isolated gastrojejunostomy versus conventional pancreaticojejunostomy group, and 700 patients in the isolated pancreaticojejunostomy + gastrojejunostomy group versus the conventional pancreaticojejunostomy group. Regarding POPF, we found no significant difference between isolated pancreaticojejunostomy and conventional pancreaticojejunostomy (OR = 0.83, 95% CI: 0.58–1.18; *P* = 0.29) (Fig. 2a) or between isolated gastrojejunostomy and conventional pancreaticojejunostomy (OR = 0.78, 95% CI: 0.42–1.42; *P* = 0.41) (Fig. 2a). One study evaluating isolated pancreaticojejunostomy + gastrojejunostomy performed by Grobmyer et al. showed that conventional pancreaticojejunostomy was associated with lower rates of POPF vs isolated pancreaticojejunostomy + gastrojejunostomy (OR = 2.90, 95% CI: 1.53–5.48; *P* = 0.001) (Fig. 2a).

#### Cr-POPF

Twelve studies provided data for the incidence of CR-POPF, with 983 patients in the isolated pancreaticojejunostomy versus conventional pancreaticojejunostomy group, and 259 patients in the isolated gastrojejunostomy versus conventional pancreaticojejunostomy group. Our meta-analysis revealed no significant difference regarding the incidence of CR-POPF between isolated pancreaticojejunostomy and conventional pancreaticojejunostomy (OR = 0.70, 95% CI: 0.35–1.42; *P* = 0.32) or between isolated gastrojejunostomy and conventional pancreaticojejunostomy (OR = 0.81, 95% CI: 0.23–2.90; *P* = 0.74) (Fig. 2b).

#### DGE

Thirteen studies provided data for the incidence of DGE, with 908 patients in the isolated pancreaticojejunostomy versus conventional pancreaticojejunostomy group, 259 patients in the isolated gastrojejunostomy versus conventional pancreaticojejunostomy group, and 80 patients in the isolated pancreaticojejunostomy + gastrojejunostomy versus conventional pancreaticojejunostomy group. We found no significant differences regarding DGE when comparing isolated pancreaticojejunostomy versus conventional pancreaticojejunostomy (OR = 1.10, 95% CI: 0.76–1.58; *P* = 0.62), isolated gastrojejunostomy versus conventional pancreaticojejunostomy (OR = 035, 95% CI: 0.11–5.20; *P* = 0.78), or pancreaticojejunostomy + gastrojejunostomy versus conventional pancreaticojejunostomy (OR = 0.99, 95% CI: 0.50–1.95; *P* = 0.96) (Fig. 2c).

#### Cr-DGE

Four studies provided data for the incidence of CR-DGE, with 239 patients in the isolated pancreaticojejunostomy versus conventional pancreaticojejunostomy group and 259 patients in the isolated gastrojejunostomy versus conventional pancreaticojejunostomy group. We found no significant differences for CR-DGE between isolated pancreaticojejunostomy and conventional pancreaticojejunostomy (OR = 2.18, 95% CI: 0.79–6.06; *P* = 0.13) or between isolated gastrojejunostomy and conventional pancreaticojejunostomy (OR = 0.36, 95% CI: 0.10–1.31; *P* = 0.12) (Fig. 2d).

### Bile leakage

**Fig. 3:**
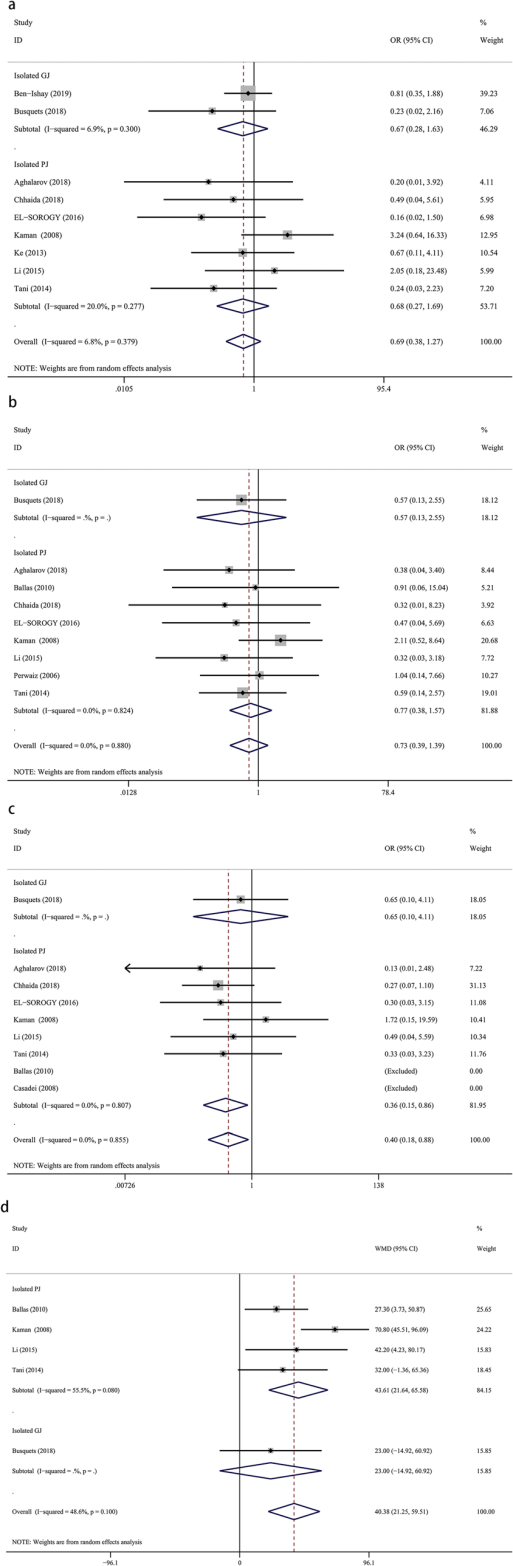
Forest plot of the meta-analysis comparing isolated pancreaticojejunostomy, isolated gastrojejunostomy, and conventional pancreaticojejunostomy regarding (**a**) bile leakage, (**b**) hemorrhage, (**c**) reoperation, and (**d**) operation time

Eleven studies provided data for the incidence of bile leakage, with 945 patients in the isolated pancreaticojejunostomy versus conventional pancreaticojejunostomy group and 259 patients in the isolated gastrojejunostomy versus conventional pancreaticojejunostomy group. We found no significant difference for the incidence of bile leakage between isolated pancreaticojejunostomy and conventional pancreaticojejunostomy (OR = 0.68, 95% CI: 0.27–1.69; *P* = 0.40) or between isolated gastrojejunostomy and conventional pancreaticojejunostomy (OR = 0.67, 95% CI: 0.28–1.63; *P* = 0.38) (Fig. 3a).

### Hemorrhage

Nine studies provided data for the incidence of hemorrhage, with 228 patients in the isolated pancreaticojejunostomy versus conventional pancreaticojejunostomy group and 80 patients in the isolated gastrojejunostomy versus conventional pancreaticojejunostomy group. We found no significant difference regarding the incidence of hemorrhage between isolated pancreaticojejunostomy and conventional pancreaticojejunostomy (OR = 0.77, 95% CI: 0.38–1.57; *P* = 0.47) or between isolated gastrojejunostomy and conventional pancreaticojejunostomy (OR = 0.57, 95% CI: 0.13–2.55; *P* = 0.46) (Fig. 3b).

### Reoperation

Nine studies provided data for the reoperation rate, with 659 patients in the isolated pancreaticojejunostomy versus conventional pancreaticojejunostomy group and 80 patients in the isolated gastrojejunostomy versus conventional pancreaticojejunostomy group. We found that isolated pancreaticojejunostomy was associated with a lower reoperation rate versus conventional pancreaticojejunostomy (OR = 0.36, 95% CI: 0.15–0.86; *p* = 0.02), but there was no significant difference between isolated gastrojejunostomy and conventional pancreaticojejunostomy (OR = 0.65, 95% CI: 0.10–4.11; *P* = 0.65) (Fig. 3c).

### Operation time

Four studies provided data for operation time, with 436 patients in the isolated pancreaticojejunostomy versus conventional pancreaticojejunostomy group and 80 patients in the isolated gastrojejunostomy versus conventional pancreaticojejunostomy group. Our results showed that conventional pancreaticojejunostomy was associated with shorter operation times versus isolated pancreaticojejunostomy (WMD = 43.61, 95% CI: 21.64–65.58; *P* = 0.00); however, there was no significant difference between isolated gastrojejunostomy and conventional pancreaticojejunostomy (WMD = 23.00, 95% CI: − 14.92–60.92; *P* = 0.23) (Fig. 3d).

### Postoperative hospital stay

**Fig. 4:**
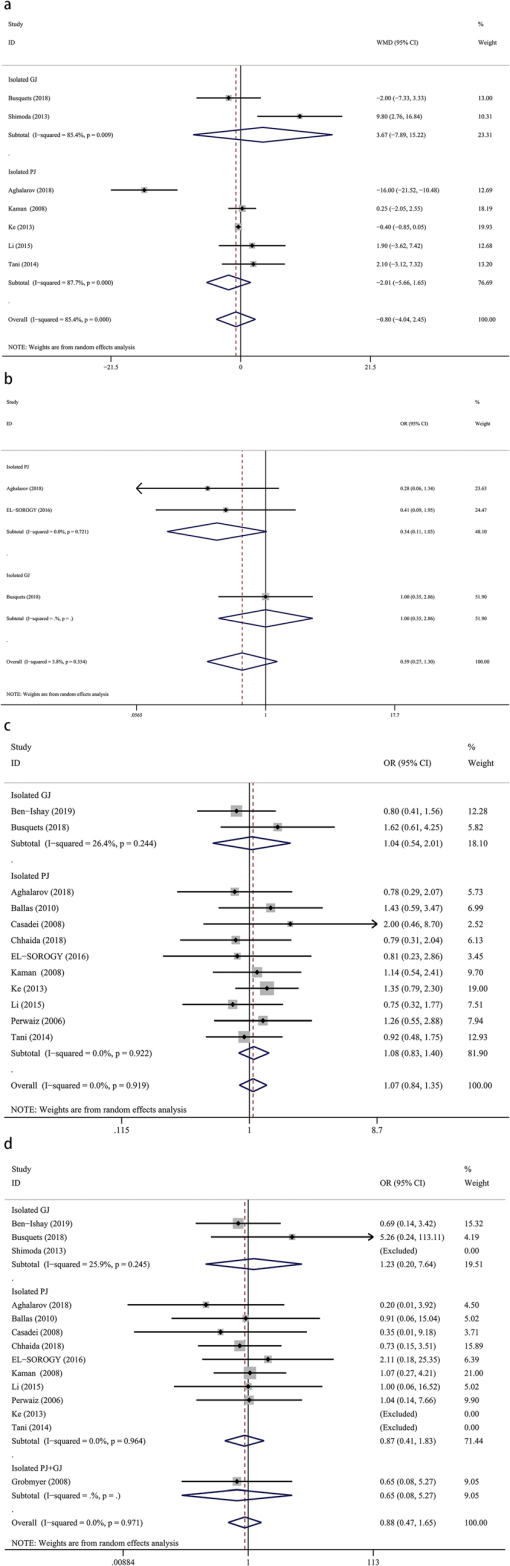
Forest plot of the meta-analysis comparing isolated pancreaticojejunostomy, isolated gastrojejunostomy, and conventional pancreaticojejunostomy regarding (**a**) length of postoperative hospital stay, (**b**) major complications, (**c**) overall complications, and (**d**) mortality

Six studies provided data for the length of postoperative hospital stay, with 659 patients in the isolated pancreaticojejunostomy versus conventional pancreaticojejunostomy group and 80 patients in the isolated gastrojejunostomy versus conventional pancreaticojejunostomy group. We found no significant difference for postoperative hospital stay between isolated pancreaticojejunostomy and conventional pancreaticojejunostomy (WMD = − 2.01, 95% CI: − 5.66–1.65; *P* = 0.53) or between isolated gastrojejunostomy and conventional pancreaticojejunostomy (WMD = 3.67, 95% CI: − 7.89–15.22; *P* = 0.28) (Fig. 4a).

### Major complications

Three studies provided data describing major complications, with 195 patients in the isolated pancreaticojejunostomy versus conventional pancreaticojejunostomy group and 80 patients in the isolated gastrojejunostomy versus conventional pancreaticojejunostomy group. Our results showed that isolated pancreaticojejunostomy was associated with lightly fewer major complications versus conventional pancreaticojejunostomy (OR = 0.34, 95% CI: 0.11–1.03; *P* = 0.05), but there was no significant difference between isolated gastrojejunostomy and conventional pancreaticojejunostomy (OR = 1.00, 95% CI: 0.35–2.86; *P* = 1.00) (Fig. 4b).

### Overall complications

Twelve studies provided data describing the overall complications rate, with 983 patients in the isolated pancreaticojejunostomy versus conventional pancreaticojejunostomy group and 259 patients in the isolated gastrojejunostomy versus conventional pancreaticojejunostomy group. Our meta-analysis revealed no significant difference between isolated pancreaticojejunostomy and conventional pancreaticojejunostomy (OR = 1.08, 95% CI: 0.83–1.40; *P* = 0.56) or between isolated gastrojejunostomy and conventional pancreaticojejunostomy (OR = 1.04, 95% CI: 0.54–2.01; *P* = 0.91) (Fig. 4c).

### Mortality

Thirteen studies provided data for mortality rates, with 983 patients in the isolated pancreaticojejunostomy versus conventional pancreaticojejunostomy group, 259 patients in the isolated gastrojejunostomy versus conventional pancreaticojejunostomy group, and 700 patients in the isolated pancreaticojejunostomy + gastrojejunostomy versus conventional pancreaticojejunostomy group. We found no significant difference in mortality rates between isolated pancreaticojejunostomy and conventional pancreaticojejunostomy (OR = 0.87, 95% CI: 0.41–1.83; *P* = 0.71), between isolated gastrojejunostomy and conventional pancreaticojejunostomy (OR = 1.23, 95% CI: 0.20–7.64; *P* = 0.82), or between isolated pancreaticojejunostomy + gastrojejunostomy versus conventional pancreaticojejunostomy (OR = 0.65, 95% CI: 0.08–5.27; *p* = 0.69) (Fig. 4d).

### Publication bias and sensitivity analysis

The funnel plots for the parameters were symmetrical, and Egger’s test revealed no significant publication bias. Sensitivity analyses were performed by removing individual studies from the data and analyzing the effect on the overall results. However, these exclusions did not alter the results.

## Discussion

This meta-analysis compared isolated pancreaticojejunostomy and isolated gastrojejunostomy with conventional pancreaticojejunostomy after pancreaticoduodenectomy. Our results showed that isolated pancreaticojejunostomy was associated with fewer major complications and lower reoperation rates, but required longer operation time versus conventional pancreaticojejunostomy. However, the rates for overall complications, POPF, CR-POPF, DGE, CR-DGE, bile leakage, and hemorrhage, and the length of postoperative hospital stay and mortality rates with isolated pancreaticojejunostomy versus isolated gastrojejunostomy were similar to rates for conventional pancreaticojejunostomy. Considering the limitations, future high-quality RCTs are required.

POPF, one of the most frequent complications after pancreaticoduodenectomy, is associated with intra-abdominal abscess, sepsis, and life-threatening hemorrhage. Many methods have been used to decrease the incidence of POPF such as using fibrin [28] or pancreatic stenting [29], and modifying the jejunal anastomosis [30]; however, the optimal technique is still debated. Our study revealed no significant difference between isolated pancreaticojejunostomy and conventional pancreaticojejunostomy, which was consistent with most previous studies. However, some studies demonstrated that isolated pancreaticojejunostomy was associated with lower rates of POPF after pancreaticoduodenectomy compared with conventional pancreaticojejunostomy [20, 31]. Several reasons were revealed in these studies, including the separation of bile acids and pancreatic enzymes [5] and decreasing the reflux of bile into the pancreas [32]; however, these advantages were theoretical, and comparative studies are lacking. Of note, adding jejunojejunal anastomosis in isolated pancreaticojejunostomy could increase intestinal intraluminal pressure, which may affect the pancreaticojejunostomy anastomosis [19]. An RCT performed by Ke et al. showed that the rate of Grade B POPF in the isolated pancreaticojejunostomy group was higher than that in the conventional pancreaticojejunostomy group [6]. In addition to the 13 studies involving 1942 patients, our study showed that isolated pancreaticojejunostomy provides no advantage over conventional pancreaticojejunostomy regarding CR-POPF. Similar to previous studies evaluating isolated gastrojejunostomy, the incidences of POPF and CR-POPF were similar to those with conventional pancreaticojejunostomy.

DGE is also one of the most frequent complications after pancreaticoduodenectomy, with rates ranging from 13.5 to 40% [14, 33]. Several surgical reconstruction procedures have been proposed to decrease the incidence of DGE, namely the Billroth I procedure, Braun enteroenterostomy, and isolated gastrojejunostomy. However, few studies have compared isolated gastrojejunostomy and conventional pancreaticojejunostomy. The debate regarding isolated gastrojejunostomy versus conventional pancreaticojejunostomy is on-going. Regarding DGE and CR-DGE, our results revealed no significant difference between isolated gastrojejunostomy and conventional pancreaticojejunostomy, and we found similar results when comparing isolated pancreaticojejunostomy and conventional pancreaticojejunostomy. A previous meta-analysis involving three studies revealed that conventional pancreaticojejunostomy was associated with lower rates of DGE versus isolated gastrojejunostomy [34]; however, the sample size in the study was small. In the current meta-analysis, we showed that the incidences of DGE and CR-DGE were similar for both isolated pancreaticojejunostomy and conventional pancreaticojejunostomy, indicating that the activation of pancreatic enzymes does not influence the occurrence of DGE.

Regarding major complications, our meta-analysis demonstrated that isolated pancreaticojejunostomy has comparable with conventional pancreaticojejunostomy. The definition of major complications in our included studies varied. Applied with Clavin-Dindo grade, there were three studies provided the data of major complications. Our study showed that there was no significantly difference between isolated pancreaticojejunostomy and conventional pancreaticojejunostomy. Interestingly, isolated pancreaticojejunostomy decreases the incidence of reoperation in this study. The study conducted by Aghalarov et al and Chhaidar et al showed that isolated pancreaticojejunostomy has less reoperation [10, 20]. They showed that the reoperation was largely because of POPF-related complications. Nevertheless, there are many factors that affect the occurrence of reoperation after surgery, such as bleeding, gastrointestinal anastomotic leakage. Additionally, with the development of percutaneous drainage and other procedures, there was fewer reoperation. However, there was lack of enough data about the detail of reoperations which may lead to bias in this present study.

## Conclusion

In conclusion, isolated pancreaticojejunostomy was associated with lower reoperation rates, but required longer operation times versus conventional pancreaticojejunostomy. The rate of major complications, overall complications, POPF, CR-POPF, DGE, CR-DGE, bile leakage, and hemorrhage, and the length of postoperative hospital stay and mortality rates with isolated pancreaticojejunostomy and isolated gastrojejunostomy were similar to the respective rates with conventional pancreaticojejunostomy. However, further randomized controlled trials are needed.

## Data Availability

All the data used in the study can be obtained from the original articles.

## Abbreviations

POPF: Postoperative pancreatic fistula
DGE: Delayed gastric emptying
CI: Confidence interval
OR: Odds ratios
WMD: Weighted mean difference
RCTs: Randomized controlled trials
